# Factors associated with different levels of daytime sleepiness among Korean construction drivers: a cross-sectional study

**DOI:** 10.1186/s12889-021-12062-3

**Published:** 2021-11-05

**Authors:** Yong Han Ahn, Sangeun Lee, Su Ryeon Kim, Jeeyeon Lim, So Jin Park, Sooyoung Kwon, Heejung Kim

**Affiliations:** 1 School of Architecture and Architectural Engineering, Hanyang University-ERICA, Room #210, Engineering II, 55 Hanyangdaehak-ro, Sangnok-gu, 15588 Ansan, Gyeonggi-do Republic of Korea; 2 College of Nursing, University of Illinois at Chicago, 845 S. Damen Ave, Chicago, IL 60602 USA; 3 College of Architecture, Texas A&M University, College Station, Texas, TX 77840 USA; 4 College of Nursing, Yonsei University, 50-1 Yonsei-ro, Seodaemun-gu, Seoul, Republic of Korea; 5 Department of Smart City Engineering, Hanyang University-ERICA, Ansan, Gyeonggi-do, Republic of Korea; 6 Sejong City Center for Infectious Diseases Control and Prevention, 5F 503, 19 Horyeoul-ro (Sejong City Hall), 30150 Sejong-si, Republic of Korea; 7 Mo-Im Kim Nursing Research Institute, Yonsei University, 50-1 Yonsei-ro, Seodaemun-gu, Seoul, Republic of Korea

**Keywords:** Construction driver, Daytime sleepiness, Driving fatigue, Occupational health promotion, Safety

## Abstract

**Background:**

Commercial vehicle accidents are the leading cause of occupational fatalities and an increased risk of traffic accidents is associated with excessive fatigue, other health problems as well as poor sleep during work. This study explores individual and occupational factors associated with different levels of daytime sleepiness and identifies their association with driving risk among occupational drivers working at construction sites.

**Methods:**

This cross-sectional and correlational study adopted a self-reported questionnaire of Korean construction drivers (*N* = 492). The data were collected from October 2018 to February 2019 using a battery of six validated instruments about participants’ sociodemographic, health-related, and occupational characteristics. One-way ANOVA and multinomial logistic regression were conducted using IBM SPSS WIN/VER 25.0, with a two-tailed alpha of .05.

**Results:**

Based on the Epworth Sleepiness Scale, “moderate” (31.7%) and “severe” (10.2%) daytime sleepiness groups were identified. There were significant differences in break time, driving fatigue, depressive symptom, subjective sleep quality, physical and mental health, and driving risk among the three groups (all *p*-values < .001). Driving fatigue (Adjusted Odds Ratio [aOR] = 1.08, 1.17), depressive symptoms (aOR = 0.91, 0.98), subjective sleep quality (aOR = 1.18 in moderate only), and driving over the speed limit (aOR = 1.43, 2.25) were significant factors for determining “moderate” and “severe” daytime sleepiness groups, respectively.

**Conclusion:**

A significant number of construction drivers experience excessive daytime sleepiness; thus it is important to reduce the negative impact of driving fatigue and other factors on daytime sleepiness. Our study findings suggest that occupational health care providers should pay attention to development and implementation of health management interventions to reduce driving fatigue that incorporate the drivers’ physical, mental, and occupational factors. Professional organizations need to establish internal regulations and public policies to promote health and safety among occupational drivers who specifically work at construction sites.

## Background

Occupational health and safety is a major concern worldwide. The World Health Organization (WHO) emphasizes the importance of improving workers’ health and reducing work-related injuries [1]. Most countries have formulated policies consistent with global regulations to enhance the safety and health of workers [1–3]. Previous studies have examined integrated occupational safety and health management for employees, such as safety education, stress management, and health promotion programs [4, 5]. However, occupational drivers are still vulnerable to work-related injuries and health issues, especially sleep related problems because insomnia is also important in predicting injuries [6]. According to the Emergency Department of the University Hospital data, specific sleep related problems such as poor sleep quality, short sleep duration, and excessive daytime sleepiness are common causes of an increase in work-related injury risks [7]. For example, sleep deprivation, sleepiness and poor sleep quality was independently associated road accident among truck drivers with obstructive sleep apnea [8]. Occupational drivers’ sleep health components are uniquely related to not only occupational accidents but also individual health concerns, such as cardiovascular disease [9–13]. Therefore, occupational health care providers must understand the health vulnerability of occupational drivers and develop relevant health management programs.

Commercial vehicle accidents are the leading cause of occupational fatalities, and can be a huge economic burden on the concerned employers and industry. The Industrial Accident Prevention and Compensation Policy Bureau [14] reported that approximately half of occupational drivers are injured in vehicle accidents, which accounts for nearly half of the occupational accidents in Korea. Furthermore, the US National Institute of Occupational Safety & Health (NIOSH) [3] stated that 22,000 workers died in work-related vehicle accidents from 2003 to 2014 and that the United States (US) employers spent about US $25 billion on work-related motor vehicle crashes in 2013; US $65,000 per nonfatal injury and US $671,000 per death. Regarding the vehicle type, in Korea, construction-related vehicles are ranked second (*n* = 4.44 per 1.0 million registered vehicles) in terms of their involvement in fatal crashes [11]. Therefore, it is imperative to understand traffic accident characteristics and relevant risk factors among occupational drivers to reduce motor vehicle mortality.

In previous studies, an increased risk of traffic accidents is associated with excessive fatigue, other health problems as well as poor sleep, such as insomnia [9, 15–17]. For example, occupational drivers in the transportation industry have reported poor sleep quality on working days [17], a higher rate of chronic diseases or obesity than the adult working population [18], high levels of job stress [19], underdiagnoses of obstructive sleep apnea syndrome [20] and more depressive symptoms [21]. Several studies have reported drowsy driving as a major risk factor for motor vehicle accidents [9–11], the mortality for which is high in drivers of commercial trucks and construction-related vehicles [11]. Drowsy driving was reported to be one of the major causes (22.5%) of traffic accidents in Korea and can be considered equally harmful as drunk driving [22]. In addition, driving fatigue and daytime sleepiness are critical predictors of drowsy driving by occupational drivers [16, 23], and increase driving risks among them [9, 16, 24, 25]. Fatigue is a comprehensive indicator that reflects physical exertion, emotional distress, and physiological impairment [26]. Daytime sleepiness influences poor health and occupational risk as a core component [27], a predisposing factor [28], and a co-occurring condition [29].

Few studies have examined the different levels of daytime sleepiness in occupational drivers [9, 16] or vehicle accident victims compared to the general adult population [24, 29–31]. These studies have frequently used the Epworth Sleepiness Scale (ESS) to evaluate daytime sleepiness propensity of individuals or certain occupation groups [9, 16, 24, 32, 33]. The ESS divides the severity of daytime sleepiness into “mild (or normal),” “moderate,” and “severe” levels [32, 34, 35]. Previous studies examined the association between daytime sleepiness and motor vehicle accidents or injuries using ESS scores [24] or severity classification [16, 30]. To effectively prevent underreporting of drowsy driving accidents, multiple levels of drowsiness behind the wheel have been discussed in previous literature [36]. Some studies have reported an inverse U-shaped severity of ESS and driver injury [32]; thus, a simple summation of ESS score as a linear pattern may not reflect this phenomenon accurately.

Previous studies have examined construction drivers’ physical and emotional health concerns, such as driving fatigue, mood disorders (including depression), stress, sleep deprivation, and bodily pain [16, 17, 26]. However, few have focused on the daytime sleepiness, traffic accidents, and health conditions, particularly of construction drivers because of several reasons. First, most studies have focused on individual characteristics rather than occupational or institutional factors. Current policies emphasize that occupational safety and health promotion should consider not only individual characteristics but also organizational support; thus, a broader range of risk factors should be evaluated. Second, homogenous groups among diverse occupation types should assess the unique characteristics of each occupation [16, 29]. Third, both driving fatigue and daytime sleepiness must be included when studying this topic, as they are interrelated and could influence the occupational drivers’ health and safety. Thus, it is necessary to conduct a more comprehensive assessment of sociodemographic, health-related, and occupational characteristics when focusing on this vulnerable group.

## Methods

### Aims

This study explores individual and occupational factors associated with different levels of daytime sleepiness among occupational drivers working at construction sites. This study specifically compare driving risk among these three groups to determine the health-related factors for the groups with “moderate” and “severe” levels of daytime sleepiness.

### Study design and setting

This cross-sectional, correlational study collected the data using an online survey by following the Strengthening the Reporting of Observational Studies in Epidemiology guidelines after obtaining the approval from the institutional review board (no. Y-2018-0087) of the affiliated university. Initially, ten companies were contacted, and site directors in seven concrete mixer companies agreed to participate. They 10 to 300 workers per site (mean; M = 79.91, standard deviation; *SD* = 40.10). Participants were recruited through convenience sampling, using flyers posted in rest and dining areas at construction sites and by word-of-mouth. Participants were eligible if they were older than 18 years, had been employed as a construction driver for at least 6 months, were able to read and write Korean, and voluntarily agreed to participate. Participants were excluded if they had been diagnosed with or were currently receiving treatment for any sleep related disorder.

**Fig. 1:**
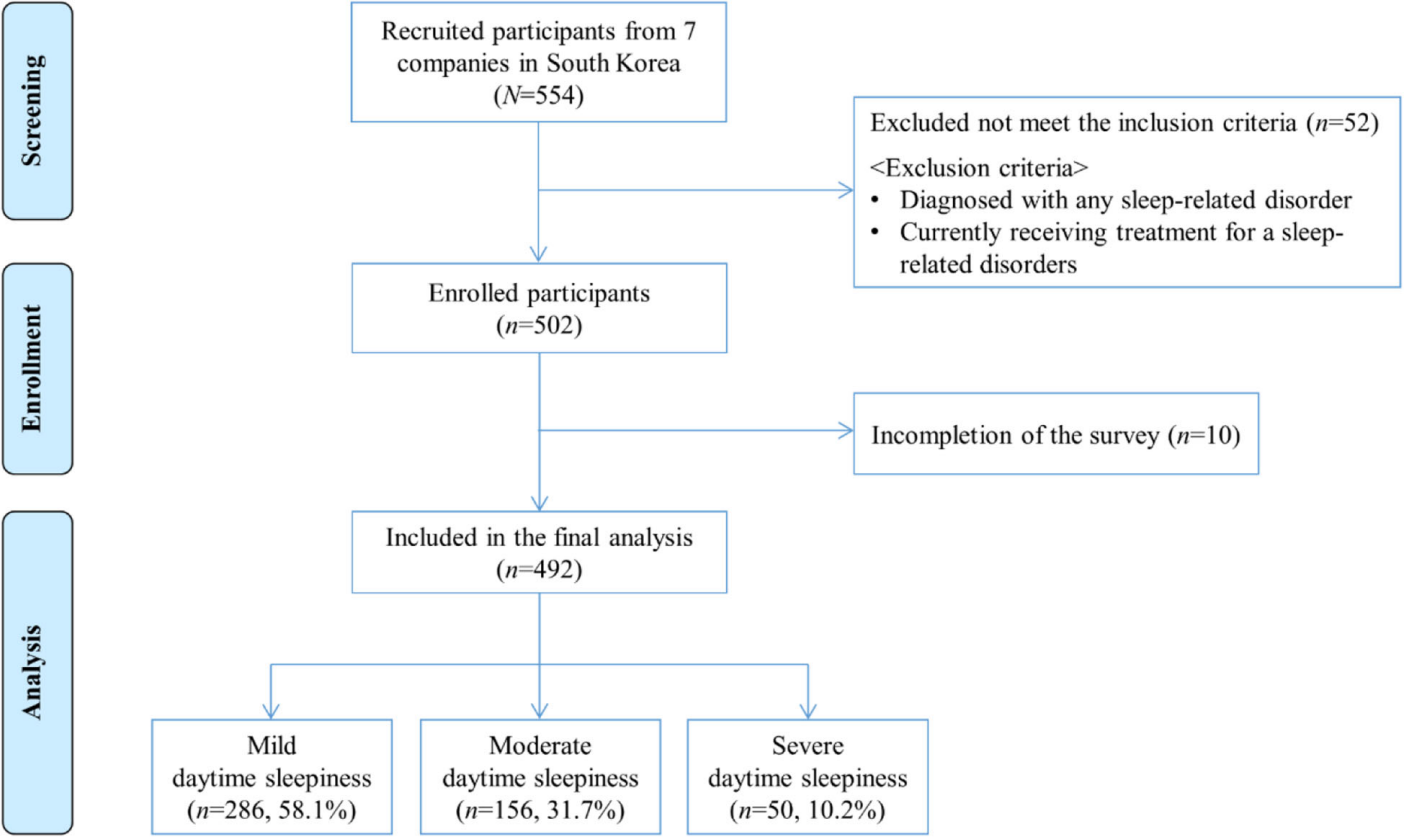
Study sample

Approximately 554 participants were recruited and screened from seven companies in South Korea, and 502 were enrolled. After excluding 10 cases, owing to incomplete responses, 492 completed questionnaires were included in the final analysis (completion rate: 98.4%) (see Fig. 1). A multivariate multinomial logistic regression analysis was conducted, based on a statistical power analysis, using the G*Power 3.1 program [37]. A minimum sample size of 442 was obtained for a logistic regression with an odds ratio (OR) = 1.4 [38], X distribution = normal, effect size = 0.04, significance set at α = .05 (two-tailed), and a power level of .80.

### Data collection and measures

Data were obtained via standardized self-reported questionnaires, from October 2018 to February 2019. All instruments for this study were provided in Korean that were evaluated as sufficiently valid and reliable in the previous studies [32, 39–43]. Participants received a small gift, amounting to US $3, as compensation for their time and effort.

### Sociodemographic and occupational characteristics

Sociodemographic characteristics included participants’ age, sex, marital status, living arrangement, education, and socioeconomic status were included. We assessed the work type, occupational health and safety insurance, work experience, and break time for the occupational characteristics.

### Health-related characteristics

Health-related characteristics included information on participants’ smoking habits, alcohol consumption, regular exercise, diagnosed diseases and number of chronic diseases, depressive symptoms, subjective sleep quality, sleep duration, physical and mental health, and daytime sleepiness. Participants reported their height and weight and the researcher calculated their body mass index.

Depressive symptoms were measured using the Korean version of the short form of the Center for Epidemiological Studies Depression Scale, developed by Radloff [44], shortened by Andresen et al. [45], and translated to Korean by Chon et al. [39]. The instrument has 10 items and uses a 4-point Likert scale; higher scores indicate more symptoms of depression (ranging from 0 to 30). The Cronbach’s α coefficients were .81 in a study of adult drivers [46] and .82 in this study.

The Pittsburgh Sleep Quality Index (PSQI) [47] was used to assess seven components of sleep quality and quantity. The Korean version of the PSQI [40] was obtained from the Mapi Research Trust on request. The global PSQI score ranges from 0 to 21, with higher scores indicating poorer sleep quality. The Cronbach’s α coefficient was .83 when the PSQI was developed [47] and it was .77 in this study.

Participants’ mental health and physical well-being were assessed using the Medical Outcomes Study 12-Item Short-Form Health Survey Version 2 [41]. The Korean version was purchased through Optum Insight Life Sciences, Inc. for use. This self-rated scale measures the components of general physical and mental health over the past 7 days (range: 0–100) [48], with higher scores reflecting a better health status. The Cronbach’s α coefficients were .67 for physical health status and .70 for mental health status [41]; they were .77 and .75 in this study, respectively.

The Korean version of the Epworth Sleepiness Scale (KESS) was used to assess daytime sleepiness. The KESS was originally developed by Johns [34] and was translated to Korean by Cho et al. [32]. The instrument includes eight items measured on a 4-point Likert scale; a higher score indicates more severe daytime sleepiness. The Cronbach’s α coefficients were .79 in a study of long-haul truck drivers in the US [49] and .82 in this study. Based on the total score, 0–9 was classified as the “mild” daytime sleepiness group, 10–14 as the “moderate” daytime sleepiness group, and 15–24 as the “severe” daytime sleepiness group.

### Driving characteristics

Driving characteristics included driving fatigue; the Traffic Accident Risk Index (TARI); dangerous driving activities, including forced driving, unfastened safety belt, driving over the speed limit, and cell phone use while driving; and vehicle accident experiences.

Driving fatigue was self-reported using the Driving Fatigue Checklist [42]. This scale includes 10 items that are rated on a 5-point Likert scale, and higher scores indicate a higher level of perceived fatigue while driving. The Cronbach’s α coefficients were .92 when the checklist was developed [42] and .93 in this study.

The TARI [43] was used to assess the traffic incident risk of the driver. It includes six items measured on a 5-point Likert scale. Risky driving activities were measured using four categories: (1) forced driving, (2) an unfastened safety belt, (3) driving over the speed limit, and (4) using a cell phone while driving [50]. The experiences of vehicle accidents were evaluated using two dichotomous questions about experiences of traffic accidents and of near misses in the past year.

### Statistical analyses

Descriptive statistics were calculated as frequencies (percentages) or means (*SD*). The chi-squared test and one-way analysis of variance were also performed along with a Bonferroni post-hoc comparison. In addition, multinomial logistic regression was conducted to identify the “moderate” and “severe” groups compared to the “mild” daytime sleepiness group. The statistical assumptions were checked, and there was no problem of multicollinearity during the regression analyses; the variance inflation factor of the independent variables ranged from 1.05 to 1.67, and the minimum tolerance was .60. The analyses were conducted using SPSS 25.0 (IBM Corp., Armonk, NY, USA), and the significance level was set at α = .05.

## Results

### Construction driver characteristics

The mean age of the participants was 53.39 years (*SD* = 9.44) and 99% were males. Approximately 80% were regular workers following a 9–5 schedule, while others worked irregularly, such as in night shifts, part-time, or on-call assignment. The average work hours per week ranged from 24 to 98 h (M = 53.68, *SD* = 10.54). Most workers drove concrete mixers at sites or trucks transporting construction materials from site to site. Table 1 provides details on participants’ sociodemographic and health-related characteristics.

**Table 1:** Differences of general characteristics among three daytime sleepiness groups

Variables	Categories	Total N (%)	Mild daytime sleepiness group n (%)	Moderate daytime sleepiness group n (%)	Severe daytime sleepiness group n (%)	*χ*^*2*^*(p*)
Age (years)	< 60	349 (72.0)	199 (70.6)	110 (71.9)	40 (80.0)	1.873 (.392)
	≥ 60	136 (28.0)	83 (29.4)	43 (28.1)	10 (20.0)	
Sex	Male	486 (99.0)	281 (98.3)	155 (100.0)	50 (100.0)	3.621 (.164)
	Female	5 (1.0)	5 (1.7)	0 (0.0)	0 (0.0)	
Marital status	Married	408 (83.4)	234 (82.4)	132 (85.2)	42 (84.0)	.568 (.753)
	Not married	81 (16.6)	50 (17.6)	23 (14.8)	8 (16.0)	
Living arrangement	Living with others	448 (91.2)	258 (90.2)	144 (92.9)	46 (92.0)	.953 (.621)
	Living alone	43 (8.8)	28 (9.8)	11 (7.1)	4 (8.0)	
Education	Middle school or under	72 (14.8)	37 (13.1)	25 (16.3)	10 (20.0)	2.453 (.653)
	High school	308 (63.4)	184 (65.0)	96 (62.8)	28 (56.0)	
	Diploma or above	106 (21.8)	62 (21.9)	32 (20.9)	12 (24.0)	
Social economic status	High	17 (3.5)	14 (4.9)	2 (1.3)	1 (2.0)	4.772 (.311)
	Moderate	297 (60.7)	168 (59.0)	96 (62.3)	33 (66.0)	
	Low	175 (35.8)	103 (36.1)	56 (36.4)	16 (32.0)	
Work type	Regular shift	381 (77.4)	228 (79.7)	113 (72.4)	40 (80.0)	3.275 (.194)
	Irregular shift	111 (22.6)	58 (20.3)	43 (27.6)	10 (20.0)	
Occupational health and safety insurance	Yes	255 (52.0)	149 (52.5)	79 (50.6)	27 (54.0)	.220 (.896)
	No	235 (48.0)	135 (47.5)	77 (49.4)	23 (46.0)	
BMI (kg/m^2^)	Normal (18.5–22.9)	112 (23.3)	76 (27.0)	26 (17.2)	10 (20.8)	7.839 (.098)
	Overweight (23.0–24.9)	137 (28.5)	77 (27.3)	50 (33.1)	10 (20.8)	
	Obese (≥ 25)	232 (48.2)	129 (45.7)	75 (49.7)	28 (58.4)	
Smoking	Current smoker	235 (47.9)	142 (49.7)	74 (47.7)	19 (38.0)	2.316 (.314)
	Not current smoker	256 (52.1)	144 (50.3)	81 (52.3)	31 (62.0)	
Drinking	Current drinker	363 (74.1)	204 (71.3)	119 (76.8)	41 (82.0)	3.351 (.187)
	Not current drinker	127 (25.9)	82 (28.7)	36 (23.2)	9 (18.0)	
Regular exercise	Yes	347 (70.9)	206 (72.0)	113 (72.9)	29 (58.0)	4.508 (.105)
	No	143 (29.1)	80 (28.0)	42 (27.1)	21 (42.0)	
Number of chronic diseases	0	293 (59.7)	183 (64.0)	79 (51.0)	31 (62.0)	7.207 (.125)
	1	156 (31.8)	81 (28.3)	60 (38.7)	15 (30.0)	
	2 or more	42 (8.5)	22 (7.7)	16 (10.3)	4 (8.0)	

Notes: missing data were excluded in analyses

### Differences in general characteristics among the three daytime sleepiness groups

The participants were classified into three groups based on the KESS scores: there were 286 participants in the “mild” daytime sleepiness group (58.1%), 156 in the “moderate” daytime sleepiness group (31.7%), and 50 in the “severe” daytime sleepiness group (10.2%).

Table 1 shows the details of the general characteristics of the three groups. The average body mass index was 25.08 (*SD* = 2.86) kg/m^2^, and 23.3% had a normal body mass index. About half of the participants (40.3%, 198/492) were diagnosed with a chronic disease, and the most common diagnosis was hypertension (22.4%), followed by diabetes, (10.0%) and gastrointestinal disease (6.7%). There was no statistical difference when comparing the general characteristics of the three groups.

### Differences in occupational and health-related characteristics among the three daytime sleepiness groups

Table 2 presents the differences in occupational and health-related characteristics among the three daytime sleepiness groups. There were significant differences in break time, driving fatigue, depressive symptoms, subjective sleep quality, and physical and mental health among the three groups. In terms of the occupational factors, participants with no daytime sleepiness reported taking a significantly longer break time while driving (*F* = 4.187, *p* = .017) and having lower driving fatigue (*F* = 52.399, *p* < .001) than the other groups, particularly the “severe” daytime sleepiness group. Regarding the health-related factors, participants with “mild” daytime sleepiness reported lower levels of depressive symptoms (*F* = 10.125, *p* < .001), better subjective sleep quality (*F* = 10.125, *p* < .001), and better physical health (*F* = 8.948, *p* < .001) and mental health (*F* = 14.389, *p* < .001) than the “moderate” and “severe” daytime sleepiness groups. Specifically, the “severe” daytime sleepiness group showed the worst levels of depressive symptoms.

**Table 2:** Differences of occupational, health-related, and driving characteristics among three daytime sleepiness groups

Variables	Categories	Mild daytime sleepiness group^a^ (*n* = 286) M (SD)	Moderate daytime sleepiness group^b^ (*n* = 156) M (SD)	Severe daytime sleepiness group^c^ (*n* = 50) M (SD)	*F (p* value)
Occupational-related factors	Driving time (minutes per week)	656.14 (90.38)	642.92 (105.76)	658.20 (115.70)	1.000 (.368)
	Break time (minutes)	74.33 (82.38)	57.96 (33.46)	65.24 (38.54)	4.187 (.017)
	Work experiences (years)	18.07 (11.07)	17.13 (10.82)	14.02 (8.96)	2.991 (.051)
	Driving fatigue	7.76 (7.16)	13.46 (7.49)	17.32 (9.04)	52.399 (<.001) a < b < c
Health-related factors	Depression	5.79 (3.90)	6.80 (4.16)	8.44 (4.88)	10.125 (<.001) a < b < c
	Subjective sleep quality	5.16 (2.84)	6.92 (2.81)	6.92 (3.00)	18.879 (<.001) a < b,c
	Sleep duration (hours)	8.35 (2.03)	8.10 (2.31)	8.36 (2.35)	.711 (.491)
	Physical health	48.10 (8.26)	46.17 (7.58)	43.70 (7.99)	8.948 (<.001) a < b,c
	Mental health	51.63 (8.26)	48.29 (9.05)	45.47 (8.88)	14.389 (<.001) a < b,c
Driving characteristics	Traffic Accident Risk Index	10.64 (4.33)	12.98 (5.02)	14.48 (4.91)	22.119 (<.001) a < b,c
	Total score of dangerous driving activity	7.82 (2.17)	8.56 (2.03)	9.72 (2.06)	19.547 (<.001) a < b < c
	Item 1. Forced driving	2.52 (1.07)	2.62 (.91)	2.88 (.92)	3.044 (.051)
	Item 2. Unfastened safety belt	1.69 (.95)	1.81 (.97)	1.76 (.94)	.833 (.435)
	Item 3. Over speed limit	1.73 (.87)	2.03 (.81)	2.54 (.93)	18.780 (<.001) a < b < c
	Item 4. Cell phone use while driving	1.87 (.73)	2.10 (.72)	2.54 (.99)	13.348 (<.001) a < b < c
	Traffic accident in the last year? Yes^§^	67 (23.4)	38 (24.4)	14 (28.0)	.489 (.783)
	Near miss accident in the last year? Yes^§^	53 (18.5)	45 (28.8)	16 (32.0)	8.470 (.014)

Note: All post-hoc tests were done by Bonferroni test. ^§^ Dichotomized as Yes vs. No

### Differences in driving risk among the three daytime sleepiness groups

There were significant differences in driving risk among the three groups. Participants with “mild” daytime sleepiness had a significantly lower TARI score (*F* = 22.119, *p* < .001) and total score for dangerous driving activities (*F* = 19.547, *p* < .001) than the other groups. The “severe” daytime sleepiness group showed the highest engagement in dangerous driving activities compared to the other groups, specifically driving over the speed limit and cell phone use while driving. Almost 23% of the participants had experienced a near miss in the past year, and more near misses were reported by the “severe” daytime sleepiness group than by the other groups (χ^2^ = 8.470, *p* < .001). Moreover, 24% had experienced at least one traffic accident in the past year, but this did not differ among the three groups (χ^2^ = .489, *p =* .783; Table 2).

### Multivariate multinomial logistic regression of the different daytime sleepiness groups

The results of the multivariate multinomial logistic regression analysis are shown in Table 3. The model explained the “moderate” and “severe” daytime sleepiness groups (Nagelkerke *R*^2^ = 0.351; referent group = “mild” daytime sleepiness group). Overall, driving fatigue, depressive symptoms, and subjective sleep quality were significantly associated with the “moderate” and “severe” daytime sleepiness groups. In addition, only one dangerous driving activity, driving over the speed limit, was found to be significant in the model (Wald *F* = 3.79, *p* = .052). The “severe” daytime sleepiness group had a higher aOR of driving fatigue (aOR = 1.17, 95% confidence interval; CI = 1.09, 1.25) than the “moderate” daytime sleepiness group (aOR = 1.08, 95% CI = 1.04, 1.13). In addition, the “moderate” daytime sleepiness group had a higher likelihood of reporting poor levels of subjective sleep quality (aOR = 1.18, 95% CI = 1.05, 1.31) than the “mild” daytime sleepiness group, whereas the “severe” daytime sleepiness group did not. Depressive symptoms were associated with the “moderate” daytime sleepiness group (aOR = 0.91, 95% CI =0.83, 0.99). Among the four dangerous driving activities, driving over the speed limit was significant, and the OR was higher in the “severe” daytime sleepiness group than in the “moderate” daytime sleepiness group (aOR = 2.25, 95% CI = 1.27, 3.98 and aOR = 1.43, 95% CI = 1.01, 2.03, respectively).

**Table 3:** Multivariate multinomial logistic regression analysis with different daytime sleepiness group

Variables (baseline)	Categories	aOR (95% CI) ^§^		Wald F(***p***)
		Moderate Daytime Sleepiness group (*n* = 120)	Severe Daytime Sleepiness group (*n* = 35)	
**Occupational-related factors**				
Break time		.99 (.99–1.00)	1.00 (.99–1.01)	3.79 (.052)
Driving fatigue		**1.08 (1.04–1.13)**	**1.17 (1.09–1.25)**	12.91 (<.001)
**Health-related factors**				
Depression		**.91 (.83–.99)**	.98 (.85–1.12)	4.97 (.026)
Subjective sleep quality		**1.18 (1.05–1.31)**	1.02 (.84–1.23)	8.47 (.004)
Physical health		1.00 (.96–1.04)	1.01 (.94–1.07)	<.01 (.970)
Mental health		.99 (.95–1.03)	.97 (.92–1.04)	.41 (.521)
**Driving-related factors**				
Traffic Accident Risk Index		1.06 (.99–1.13)	1.06 (.96–1.18)	2.63 (.105)
Each dangerous driving activity				
Item 1 Forced driving		.83 (.62–1.10)	.88 (.51–1.52)	1.74 (.187)
Item 2 Fastened safety belt		1.11 (.84–1.48)	1.07 (.67–1.73)	.56 (.455)
Item 3 Over speed limit		**1.43 (1.01–2.03)**	**2.25 (1.27–3.98)**	3.98 (.046)
Item 4 Cell phone use while driving		1.16 (.79–1.71)	1.65 (.89–3.06)	.58 (.447)
Experiences of traffic accident (ref. No)	Yes	.63 (.34–1.15)	.61 (.23–1.64)	2.26 (.133)
Experiences of near miss accident (ref. No)	Yes	.93 (.49–1.77)	.58 (.21–1.63)	.05 (.829)
Nagelkerke R^2^		.351 (<.001)		

^§^Logistic regression with complex sample was conducted with no daytime sleepiness group as a referent

## Discussion

The current study sought to examine the individual and occupational factors related to daytime sleepiness levels and identified their association with the driving risk of commercial drivers working at construction sites. Several occupational and health-related factors were significantly associated with higher levels of daytime sleepiness among the construction drivers, including driving fatigue. Approximately 40% of construction drivers had at least “moderate” levels of daytime sleepiness.

Higher levels of driving fatigue were related to “moderate” and “severe” daytime sleepiness. In this study, driving fatigue was highest in the “severe” daytime sleepiness group, followed by the “moderate” daytime sleepiness group and “mild” daytime sleepiness group. There is supporting evidence of a strong association between daytime sleepiness and fatigue, which play significant roles in exacerbating the occurrence of car crashes similar to the previous studies [9, 10, 16, 24, 25]. The US Department of Transportation established the National Highway Traffic Safety Administration (NHTSA) strategic plan to control fatigue and daytime sleepiness and ensure driver safety by implementation of specific features, such as vehicle warning systems and roadway rumble strips [51]. Occupational healthcare providers should offer comprehensive health promotion programs, such as advising about healthy sleep habits and work schedules, and employers should make an effort to reduce excessive fatigue at work, by preventing long working hours or irregular work schedules as recommended [33]. For example, in Korea, there are approximately 300 rest areas built 25 km apart and the government plans to construct additional service areas for resting on the expressways and national highways [52]. The government also needs to provide regular medical checkups for construction drivers, with sleep problems, that threaten their safety while driving.

Interestingly, participants who had experienced more depressive symptoms in the past 12 months were less likely to experience “moderate” daytime sleepiness, but this was not found in the “mild” daytime sleepiness or “severe” daytime sleepiness groups. In general, depression is known to involve physical and emotional factors that affect daytime sleepiness as consistent to the previous literature [9, 16]. However, the multivariate regression analysis were unexpected because the relative association between depressive symptoms and daytime sleepiness becomes weaker when considering the impact of other factors. In general, the relationship between depression and sleep is bidirectional and complicated [53]. Since depression is known to result in both sleep deprivation and excessive sleep [53], the effect of depression on daytime sleepiness may be inconsistently shown the following day.

Recent studies reported that the pathophysiological mechanisms of depression induce stable arousal during the day [54, 55], which decreases daytime sleepiness. Future studies should reconsider the choice of instruments to measure depression. The measure of depressive symptoms used in this study was developed to assess the experience of depression and is highly focused on the general public’s negative emotions [39]. However, daytime sleepiness and driving fatigue are considered more as physical symptoms [16]. Thus, it is possible that the relationship between depressive symptoms and daytime sleepiness was not clearly revealed in this study. Considering the complex nature of depression and sleep disturbances, a deeper examination of these two conditions is necessary, using different measures and by focusing on the conceptual framework of depression and other comorbidities.

In contrast, higher levels of subjective sleep quality were associated with “moderate” daytime sleepiness. It is important to consider the effect of both sleep quantity and quality on health conditions [17]. Previous studies reported that sleep quality is related more to the safety issues or occupational performance of drivers than sleep duration [17, 25]. For example, the group with poor sleep quality reported higher levels of daytime sleepiness and daytime fatigue even after having the same amount of sleep [31]. In addition, the occupational drivers’ irregular work schedules can result in poor sleep quality which is related to drowsy driving. Occupational health providers should educate occupational drivers about proper sleeping habits compensating for insufficient sleep, and allow access to medication, if necessary.

We also investigated the differences in driving characteristics among the daytime sleepiness groups. There were significant differences in the global traffic accident risk and number of near misses in the past year. The higher the level of daytime sleepiness, the higher the risk of car accidents and the higher the number of near misses. However, it was not significantly associated with traffic accidents in the past year, possibly due to the limitation of self-reporting and other biases as similarly found in the literature [56]. Daytime sleepiness is associated with car accidents that threaten the safety of drivers as consistent to the previous studies [9, 10, 15–17, 24, 25]. Therefore, daytime sleepiness should be properly controlled through individual efforts, implementation of environmental improvement initiatives by the company, and following of public health regulations [57]. Occupational drivers are particularly vulnerable to daytime sleepiness [25]. Consequently, a medical check-up of excessive daytime sleepiness, which considers other comorbidities, such as obesity or obstructive sleep apnea [58], is highly recommended for occupational drivers.

We also found that dangerous driving activities were significantly different according to the level of daytime sleepiness. The “severe” daytime sleepiness group reported higher levels of forced driving, driving over the speed limit, and cell phone use compared to the other groups. Specifically, driving over the speed limit was a key activity that was associated more with the “severe” daytime sleepiness group and it was greater than even in the “moderate” daytime sleepiness group. These findings support previous studies that focused on the association between sleepiness and hazardous behavior, including over speeding or using a cell phone [16, 24]. Bener et al. [24] reported that drivers who had vehicle accidents were less likely to use a safety belt, comply with the regular speed limit when driving, and had higher scores of daytime sleepiness than drivers who did not have vehicle accidents. In addition, occupational drivers tend to violate regulations when they feel exhausted and drowsy while on duty [59]. Similar to a Chinese study, such illegal and hazardous driving behaviors could predispose them to injuries while driving [60]. It is very important to prevent job-related injuries, considering that almost half (48%) of the drivers who went over the speed limit wear not wearing a safety belt, while drivers who conformed with the regular speed limit account for 21% of the total number of fatal crashes in the US [61]. One of the most effective ways to prevent vehicle crashes by complying with safety regulations is to promote an altruistic attitude, foster social support from others, create safety campaigns, and provide education about traffic violations using mobile phone [36, 59, 62, 63] to prevent risky behaviors while driving.

Our findings also showed that there were significant differences in break time among the three different daytime sleepiness groups. This coincides with the findings of Chen and Xie [64] about the importance of a short break while driving. According to research on long-haul drivers, working overtime negatively affects driving-related factors, such as concentrating while driving and driving skills [17]. There is evidence that insufficient rest can cause drowsy driving as well as vehicle crashes related to drowsy driving [15]. It has also been reported that half of the occupational drivers suffer from chronic fatigue and this is associated with a short break time and excessive work-related stress [19]. In 2017, the Korean government announced the enforcement of a presidential decree to take a mandatory 30-min break after every 4 h of driving to reduce daytime sleepiness and fatigue [65]. Therefore, formulation of punitive policies is highly recommended when mandatory education for drivers is not provided. Constant surveillance should also be conducted to reduce the physical and psychological burden while driving. In addition, it is important to develop more effective interventions that could be used during a short break. For example, a recent study showed the effectiveness of a relaxation technique during naps to improve occupational performance of the drivers’ duties [66]. Therefore, occupational health providers should educate construction drivers using specific strategies or evidence-based interventions.

### Limitations and suggestions for further study

This study has several limitations and recommendation for the further research. First, the cross-sectional, correlational design of this study limits the determination of causality between the construction driver’s variables, the daytime sleepiness levels and driving risk outcomes. We suggest a longitudinal study with diverse types of construction drivers to identify the mentioned causality. Second, due to the small sample size and inclusion of only Korean participants, our study findings are not generalizable to other population. Further studies should use probability sampling and replicate the study at more sites in other countries. Furthermore, our data collection relied on the self-report of the participants’ subjective concerns regarding driving fatigue, daytime sleepiness, depression, and the experience of past traffic accidents. Since each driver has varying occupational conditions, including work shifts and different vehicle types and mileage, future research studies should objectively measure the study variables by including more information about the occupational environment.

## Conclusion

A significant number of construction drivers experience excessive daytime sleepiness, which is associated with health problems and occupational risk. It is important to reduce the negative impact of driving fatigue and other factors on daytime sleepiness. Our study findings suggest that occupational health care providers should pay attention to development and implementation of health management interventions to reduce driving fatigue that incorporate the drivers’ health-related characteristics. Specifically, occupational health care providers should provide integrated health promotion programs for drivers working at construction sites dealing with physical, mental, and occupational factors. Professional organizations need to establish internal regulations and public policies to promote health and safety among occupational drivers who specifically work at construction sites.

## Data Availability

The datasets used and/or analyzed during the current study are available from the corresponding author on reasonable request.

## Abbreviations

aOR: adjusted Odds Ratio
BMI: Body mass index
CI: confidence interval
ESS: Epworth Sleepiness Scale
KESS: Korean version of the Epworth Sleepiness Scale
M: Mean
NHTSA: National Highway Traffic Safety Administration
NIOSHI: National Institute of Occupational Safety & Health
OR: Odds Ratio
PSQI: Pittsburgh Sleep Quality Index
ref.: reference of each independent variable group
SD: standard deviations
TARI: Traffic Accident Risk Index
US: United States
WHO: World Health Organization
